# Comparing the Effect of Preoperative Administration of Methylprednisolone and its Administration Before and During Surgery on the Clinical Outcome in Pediatric Open Heart Surgeries

**DOI:** 10.5812/ircmj.8105

**Published:** 2013-06-05

**Authors:** Ghasem Soltani, Mohammad Abbasi Tashnizi, Ali Asghar Moeinipour, Mahmoud Ganjifard, Jamil Esfahanizadeh, Alireza Sepehri Shamloo, Seyed Javad Purafzali Firuzabadi, Nahid Zirak

**Affiliations:** 1 Department of Anesthesiology, Imam Reza Hospital, Mashhad University of Medical Sciences, Mashhad, IR Iran; 2 Department of Cardiac Surgery, Imam Reza Hospital, Mashhad University of Medical Sciences, Mashhad, IR Iran

**Keywords:** Cardiopulmonary Bypass, Heart Defects, Inflammation, Congenital

## Abstract

**Background:**

Cardiac surgery under Cardiopulmonary bypass causes a systemic inflammatory response with a multifactorial etiology including direct tissue damage, ischemia and stimulation of immune system induced by cardiopulmonary bypass. This study was designed due to the high prevalence and complications of this stimulated immune system in mortality, morbidity, length of ICU stay, and mechanical ventilation.

**Objectives:**

This study was aimed to compare preoperative and intraoperative methylprednisolone (MP) to intraoperative MP alone with respect to postbypass inflammation and clinical outcome.

**Patients and Methods:**

Sixty pediatric patients (age < 5years) undergoing cardiopulmonary bypass surgery between September 2011-2012 at Imam Reza hospital-Mashhad were randomly assigned to receive preoperative and intraoperative MP (group 1: 30 mg/kg, 4 hours before bypass and in bypass prime, n = 30) or intraoperative MP only (group 2: 30 mg/kg, n = 30). Postoperative temperature (peak temperature and average temperature during the first 24 hours), amount of inotropic, duration of mechanical ventilation, ICU stay, WBC, BUN, creatinine, and CRP were recorded and compared in both groups. Data were analyzed with SPSS version 13 by T-test, Mann-Whitney test if necessary, and Chi-squared distribution.

**Results:**

Patient characteristics including age, weight, gender, and duration of bypass were almost similar in both groups (P > 0.05). No significant difference in amount of inotropic medications used for hemodynamic supports, duration of mechanical ventilation, peak and average temperature and length of ICU stay was observed. Among the laboratory tests (WBC, BUN, creatinine, CRP) only WBC counts raised more in group 2 when compared to group 1(P < 0.05).

**Conclusions:**

There was no difference in clinical outcome after cardiac surgery when we administered an additional dose of methylprednisolone compared to a single dose of methylprednisolone.

## 1. Background

Correction and palliation of some congenital heart diseases requires the use of cardiopulmonary bypass (1). Cardiac surgeries under cardiopulmonary bypass induce the systemic inflammatory response with a multifactorial etiology, including direct tissue damage, ischemia, and stimulation of the immune system by CPB cycle (2).This inflammatory response is very similar to the inflammatory response in sepsis, burns and multiple traumas, and it damages target organs (2). Inflammatory effects followed by CPB are known and prevalent; and 25% of patients with LCOS (Low cardiac output syndrome) after this procedure (3). CPB- dependent inflammation is the initial cause for tissue damage and impaired heart, kidney and other organs performance (4). The amount of tissue damage depends on inflammation response to some degree. Therefore, different strategies for reducing the inflammatory response are to be used (5). For a long time, steroids were used to reduce the inflammatory response and they are well-known for their anti-inflammatory effects. Although steroids reduce inflammatory cytokine and increase anti-inflammatory cytokine, and improve heart performance, in some studies, clinical improvement including reduced need for fluid therapy, fever reduction, did not show shorten hospital stay and mechanical ventilation with steroids (6-8). Noting the peak effect of methylprednisolone is 1 to 4 hours after its administration and the duration of action is 12 to 24 hours, it seems that an extra dose of methylprednisolone given one to four hours before the operation (in addition to routine intraoperative dose) inhibits the inflammatory reaction more than the intraoperative dose (9-11). We administered a single dose of methylprednisolone based on our local protocol. The clear point is that this inflammatory response after surgery affects clinical signs caused by the systemic inflammation including length of stay in ICU, duration of mechanical ventilation, hemodynamic status, and heart dysfunction. Therefore, determination of steroid effects and different methods of its prescription is necessary in response reduction, results improvement, and finding a way to reduce the inflammatory response to reduce deaths.

## 2. Objectives

The purpose of this study is to determine and compare clinical signs of systemic inflammatory response after cardiopulmonary bypass in patients receiving multidose methylprednisolone (prior and during surgery) with patients receiving mono-dose methylprednisolone (during the surgery).

## 3. Materials and Methods

This study was performed as a double-blind clinical trial from September 2010 to September 2011 on 60 children younger than 5 years who underwent cardiopulmonary bypass (CPB) for repair of congenital heart disease in Open Heart Surgery of Imam Reza hospital in Mashhad. The trial was performed in accordance with the declaration of Helsinki and approved by the ethic committee at Mashhad University of Medical Sciences. After explaining this research to supervisors of patients, consent form and information sheet were completed by them, and then the patients were enrolled in the research.

Patients with corticosteroid allergy, chronic corticosteroid recipients, those who had received Aprotinin during surgery, patients with upper airway infection, and patient with sepsis and diabetes, patients with GI bleeding and cardiac arrest in a week before the operation were excluded. Determining of sample size was performed by using results of Valerie et.al study (1), 30 people were considered for each group. Sampling was conducted in two stages, first stage nonrandom, based on objective and second stage in a random allocation. Thus, the first patient was placed in group A (case group), and then patients were placed in each of the two groups one by one.

In mentioned manner patients were placed in two groups of multidose methylprednisolone (case group: before and during surgery) and mono-dose methylprednisolone (control group: during surgery). The case group received 30mg/kg intravenous methylprednisolone 4 hours before the operation, and they also received the same amount of drug in prime liquid during the beginning of the operation. Control group just received 30 mg/kg methylprednisolone during the beginning of the operation in prime liquid. Patients were placed under general anesthesia by fentanyl, muscle relaxants, and isoflurane. For circulatory support full-flow bypass with relative hypothermia was used. During aorta cross- clamp Cold-blood cardioplegia with additional dosing at 20- to 30-minute intervals was used. Ultra filtration was used in all cases. After the operation, all patients were ventilated with a volume of 10-15 mL / kg, and the ventilation rate to control Pco2 was matched at 35 mm hg. Fluid therapy was monitored by the anesthesiologist, and diuretic began the day after the operation.

Postoperatively the peak and average 24 hours fever were measured in the ICU (rectal temperature was measured every hour and the average 24-hour was calculated based on this), duration of ventilation, length of stay in ICU, and the rate of drug Inotrop use within 24 hours after the operation were checked, and the two groups were compared. Regarding that the above mentioned cases show the amount of tissue damage and inflammatory response, significant difference in emergence of these effects shows the different effects of the two types of methylprednisolone administration on clinical outcomes of this surgery. Aortic clamping time (minutes), time of CPB (minutes), time of ventilation (h), the amount of prescribed Inotrop, the dosage of prednisolone, duration of hospitalization in ICU, temperature, experimental data such as: White blood cell (WBC), Blood urea nitrogen (BUN), Creatinine (Cr), and C-reactive protein (CRP) and information such as age, weight and sex were also recorded by one of the nurses. And all operation were performed by one surgeon, and all experiments were performed by a specialist of laboratory science.

After surgery, all obtained data were collected and entered SPSS ver11.5 software. To study homogeneous distribution for age, sex, cardiopulmonary (CPB) and aortic clamp time, ANOVA was used in the two groups, and these variables were described by indices, charts and tables. In this analysis at first, normality of quantitative variables was determined by Kolmogorov- Smirnov test, in which among variables, hospital stay, and dose of administrated Inotrop did not follow normal distribution but ventilation time, and temperature followed the normal distribution. In the case of normality, T-test was used and otherwise Man-Whitney test was used for comparison. The significance level in this study was considered 0.05.

## 4. Results

60 children (25 females and 35 males) younger than 5 with congenital heart diseases, who underwent elective heart surgery under cardiopulmonary bypass, were randomly placed in two groups; mono-dose prednisolone (n = 30) and multidose prednisolone (n = 30). On the basis of ANOVA, the two groups did not show significant differences in mean age, weight, sex, CPB time, and aorta clamping. The two groups were homogeneous (Table 1). 

**Table 1.** Characteristics of Patients Underwent Cardiopulmonary Bypass (CPB) for Repair of Congenital Heart Disease

	Multi-dose group, n = 30	Mono-dose group (n=30)	P Value
**Age, y**	40.1 ± 18.0	39.5 ± 18.2	NS
**Gender **			
Male	18	12	NS
Female	17	13	NS
**Weight, Kg**	25.6 ± 12.4	30.6 ± 9.8	NS
**CPB time, min**	32.2 ± 9.7	32.2 ± 12.5	NS
**Ventilation time, h**	8.6 ± 7.1	12.7 ± 7.9	NS
**Aortic cross-clamp time, min**	23.1 ± 12.1	22.8 ± 13.1	NS
**The amount of prescribed Inotrop **	290.5 ± 201.2	209 ± 204.5	NS

Patients who had received mono-dose prednisolone, compared to the patients who had used multidose prednisolone, had more ICU stay, although this difference was not significant (2.15 vs. 2.02 days, P = 0.59). The mean body temperature and maximum mean body temperature in multidose patients after surgery were more than patients with mono-dose within 24 hours. However, none of the mentioned differences were statistically significant (P = 0.44, P = 0.40) (Table 2). 

**Table 2.** Clinical Changes of Patients Underwent Cardiopulmonary Bypass (CPB) for Repair of Congenital Heart Disease

	Multi-dose, n=39	Control group, n=40	P Value
**WBC^a^, 10.8 x 109/L**			
Before surgery	9.3 ± 2.6	8.8 ± 1.8	NS
After surgery	14.6 ± 5.0	13.0 ± 3.8	NS
**BUN^a^, mg/dL**			
Before surgery	17.0 ± 6.4	18.6 ± 7.7	NS
After surgery	18.5 ± 7.6	18.1 ± 6.3	NS
**Creatinine, mg/dL**			
Before surgery	0.5 ± 0.1	0.5 ± 0.1	NS
After surgery	0.6 ± 0.1	0.6 ± 0.2	NS
**C-reactive protein, mg/L**			
After surgery	2.5 ± 1.2	4.3± 4.7	NS
**Mean body temperature, C**			
After surgery	37.3 ± 0.5	37.2 ± 0.4	NS
**Maximum mean body temperature, C**			
After surgery	38.2 ± 0.6	38.2 ± 0.5	NS

^a^ Abbreviations: WBC, white blood cell; BUN, blood urea nitrogen

Two groups were not significantly different in laboratory criteria including WBC, BUN, creatinine, before and after the intervention and CRP after the intervention (Table 2). In the multidose group, WBC after the operation was more than mono-dose group (14.6 vs. 13.0, P = 0.18). Of course this level was reported in multidose group significantly higher than mono-dose group before the operation, (9.3 vs. 8.8, P = 0.32). However, in the mono-dose group, taking a dose of prednisolone significantly increased the level of WBC after the operation (P = 0.00), while in multidose group with increasing WBC, this value was not statistically significant (P = 0.18). Although before surgery BUN level in the multidose group was lower than the mono- dose group (17.0 vs. 18.6, P = 0.41), but at 24 hours after the operation, BUN level in the multidose group was higher than the mono- dose group. However, in multidose group the intervention of two doses prednisolone before and during the operation significantly increased the BUN level after the operation (P = 0.05), while in mono-dose group, with increasing BUN this value was not statistically significant (P = 0.48). Creatinine levels after the operation in multidose groups was increased more than the mono-dose group (P = 0.76). After the operation, CRP had a higher level in the multidose group compared to the mono-dose group (4.3 vs. 2.5, P = 0.10). 

Mono-dose group needed longer mechanical ventilation (12.70 ± 7.9 hours vs. 8.66 ± 7.1 for the multi-dose group), but these differences were not statistically significant (P = 0.71). During this study, all patients were discharged from hospital with no unusual signs. In none of the patients, signs of drug therapy such as postoperative bleeding, hypertension, gastrointestinal bleeding, and severe water and electrolytes disorders were observed, and all patients completed the study.

## 5. Discussion

In our study the comparative analysis was performed between the two types of mono-dose and multidose methylprednisolone (30 mg/per kg) and its impact on clinical outcome after the operation was studied on the two groups of 30 children younger than 5 years old with congenital heart disease undergoing open heart surgery (cardiopulmonary bypass). Considering that the peak of methylprednisolone effect is 1 to 4 hours after the administration, the idea of an additional dose methylprednisolone administered one hour before the operation (in addition to routine intraoperative dose) with the possibility of more effects on inhibiting the inflammatory reactions has provided the motivation for performing this investigation in minds of previous and this study researchers. A study similar to us was conducted by Dr. Valerie et al. (1) on 29 patients who were divided into two groups (14 cases of multidose and 15 cases in mono-dose group). Given the limitations of their study's sample size as well as statistical analysis, our study was conducted on more patients (two groups of 30 persons). As Valerie et al study, in our study the two groups were homogeneous in age, sex, duration of aortic clamping time, and weight.

In Valerie et al study, patients who received multidose methylprednisolone, had shorter ICU stay (4/4 days versus 6/1 day), although it was not statistically significant. In another study by Eric M. Graham et al. (12) on 76 patients (39 multidose patients and 37 mono-dose patients), there was also no difference between the two groups in the ICU and hospital same as our study. Another study by Bronicki et al. (13) showed that patients who received dexamethasone (1 mg / kg) compared to a group that received normal saline one hour before CPB had less ICU stay. In Valerie study, the average temperature and peak temperature were clearly less than mono-dose group in multidose group; but in our study there was no significant difference in peak temperature and the average temperature of mono-dose and multidose groups.

Preoperative laboratory amounts including WBC, BUN, and creatinine were homogeneous in the two groups; but in the statistical analysis performed to compare changes in the laboratory indices after surgery and before it, between the two groups, a significant increase was observed in WBC in mono-dose group, this increase in multidose group was not significant. CRP levels were compared after the operation in the two groups, though the level was higher in mono-dose group, but it was not significantly different with multidose group. In the study by Eric M. Graham et al. serum creatinine in multidose group was significantly higher compared to the mono-dose group (0.6 ± 0.1 mg /dl vs 0.5 ± 0.1 mg /dl, P = 03). In the study by Bronicki et al. the number of patients receiving dexamethasone whose serum creatinine has increased 0.2 mg/dl or more than before the operation, was significantly lower in comparison to control group (1 of 15 versus 7 of 14; P = 0.014).

In our study, like Valerie study, no cases were observed of dehiscence and wound infection, resistant hyperglycemia, and sustained hypertension. In this study, a control group that did not receive methylprednisolone was not available to compare with these two groups, so it is recommended to compare these three groups in future studies. Although a high percentage of patients in our study had ASD and VSD, but because of the diversity of these congenital heart disorders and their association with other heart disorders, including PS, TOF, PDA, with different intensity and congenital heart disease association with other genetic and chromosomal structure disorders which creates a specific background for patients, the precise integration of the patients was not possible in this regard.

It is recommended to perform further investigations with a more limited type of heart disease (only 2 or 3 common types), more careful selection of patients based on preoperative status, the type of disorder and pulmonary hypertension, and presence or absence of cyanosis. Quality and quantity of repaired organs and the extent of the deficiencies that are remained have direct and obvious effects on patients’ clinical outcomes, therefore, in the future studies; these criteria should also be considered for the comparison of groups In summary, in our study the clinical criteria including length of stay in ICU, duration of mechanical ventilation, peak and average temperatures, inotrop need and the laboratory criteria including CRP, BUN, and creatinine were of minor importance and worthless statistically. WBC Increase in mono-dose group was significantly higher than multidose group. Thus, currently administration of multidose methylprednisolone as routine is not recommended.
